# Principle of contrast-enhanced ultrasonography

**DOI:** 10.1007/s10396-024-01443-x

**Published:** 2024-05-23

**Authors:** Yoshitaka Mine, Etsuo Takada, Katsutoshi Sugimoto, Fuminori Moriyasu

**Affiliations:** 1 Department of Radiological Sciences, International University of Health and Welfare, 2600-1 Kitakanemaru, Otawara, Tochigi 324-8501 Japan; 2 Center of Medical Ultrasonics, Dokkyo Medical University, Mibu, Tochigi Japan; 3 Department of Gastroenterology and Hepatology, Tokyo Medical University, Tokyo, Japan; 4 Center for Cancer Ablation Therapy, Sanno Hospital, International University of Health and Welfare, Tokyo, Japan

**Keywords:** Microbubbles, Contrast-enhanced ultrasound, Contrast agents, Nonlinear imaging, Sonazoid

## Abstract

Sonazoid, an ultrasound contrast agent, has been covered by insurance in Japan since January 2007 for the diagnosis of hepatic mass lesions and is widely used for diagnosing not only primary liver cancer but also liver metastases such as those from breast cancer and colorectal cancer. Contrast-enhanced ultrasound for breast mass lesions has been covered by insurance since August 2012 after phase II and phase III clinical trials showed that the diagnostic performance was significantly superior to that of B-mode and contrast-enhanced magnetic resonance imaging. This paper describes the principles of imaging techniques in contrast-enhanced ultrasonography including the filter, pulse inversion, amplitude modulation, and amplitude-modulated pulse inversion methods. The pulse inversion method, which visualizes the second-harmonic component using the nonlinear scattering characteristics of the contrast agent, is widely used regardless of the contrast agent and target organ because of its high resolution. Sonazoid has a stiffer shell and requires a higher acoustic amplitude than Sonovue to generate nonlinear vibrations. The higher transmitted sound pressure generates more tissue harmonic components. Since pulse inversion allows visualization of the tissue harmonic components, amplitude modulation and amplitude-modulated pulse inversion, which include few tissue harmonic components, are primarily used. Amplitude modulation methods detect nonlinear signals from the contrast agent in the fundamental band. The mechanism of the amplitude modulation is considered to be changes in the echo signal’s phase depending on the sound pressure. Since the tissue-derived component is minor in amplitude modulation methods, good contrast sensitivity can be obtained.

## Introduction

Many contrast agents have been developed for contrast-enhanced ultrasound, and clinical trials have been conducted. Ultrasound is reflected at the boundaries between media with different acoustic impedance (the product of the speed of sound and density of the medium), and the greater the difference, the more efficiently the ultrasound is reflected. This makes gas the most suitable material for contrast agents.

Ultrasound contrast agents have been developed as microbubble formulations that can be administered intravenously. In order to contrast target organs via a peripheral vein, the microbubbles must pass through the pulmonary capillary bed and not disintegrate when exposed to left ventricular pressure. In addition, the particle size must be the same size or smaller than a red blood cell.

Sonovue (LUMASON), Definity (Luminity), Optison, and Sonazoid are widely used in clinical practice [1]. Nycomed-Amersham began developing Sonazoid in Norway in the 80 s. In Japan, Daiichi Sankyo Company, Limited began conducting phase I clinical trials in 1998, phase II trials in 1999, and phase III trials in 2001. Following these, it conducted an exploratory study in 2003 to investigate its efficacy for evaluation after radiofrequency ablation therapy of liver cancer. In January 2007, insurance coverage was extended to hepatic mass lesions, for which it has excellent qualitative diagnostic accuracy. It is widely used for diagnosing not only primary liver cancer but also liver metastases such as those from breast cancer and colorectal cancer [2]. Contrast-enhanced ultrasound for breast mass lesions has been covered by insurance since August 2012 [3–5] because of its significantly superior diagnostic performance compared to B-mode ultrasound and contrast-enhanced magnetic resonance imaging (MRI) in phase II and phase III clinical trials. Sonazoid is currently manufactured by GE HealthCare. The coverage area has been expanded to Korea, Taiwan, and Norway.

This paper describes the basic characteristics and administration method of Sonazoid used in the mammary region. We then describe the principles of imaging methods for contrast agents, including pulse inversion, amplitude modulation, and amplitude-modulated pulse inversion. In particular, the imaging methods with pulse inversion and amplitude modulation used in the mammary region will be explained using both simulation results and experimental data.

## Ingredients and characteristics of Sonazoid formulation

**Fig. 1:**
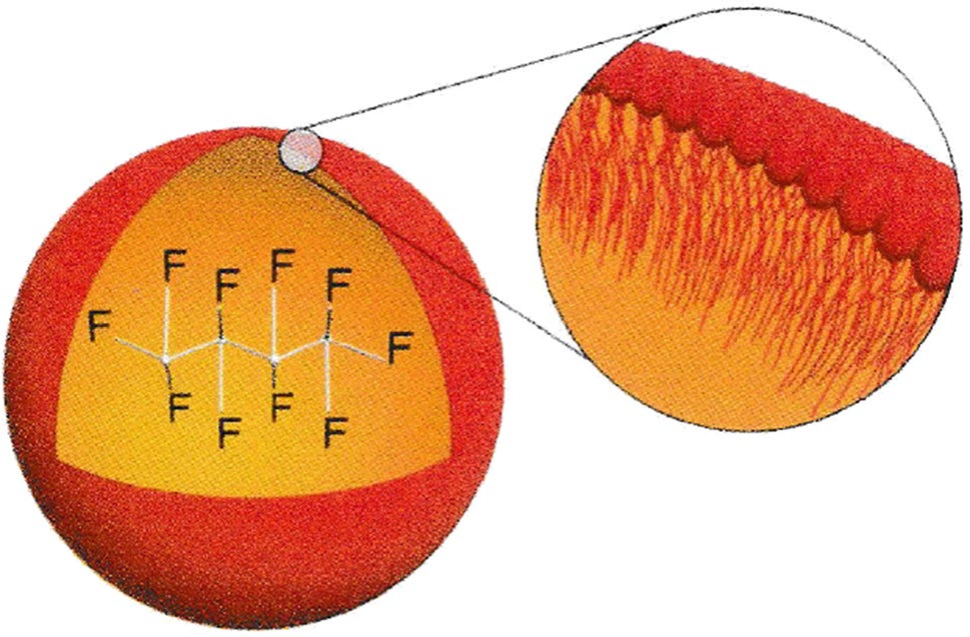
The physicochemical structure of Sonazoid microspheres; PFB stabilized with a monomolecular membrane of HEPS 2 to 3 nm thick. Adapted from Ref. [8]

The active ingredient of Sonazoid is perflubutane (C4F10) (PFB) microbubbles. PFB is gas stabilized with sodium hydrogenated egg yolk phosphatidylserine (H-EPSNa) (Fig. 1). PFB is chemically stable and water-insoluble, making it difficult to dissolve in blood in vivo. The membrane component of the PFB microbubble is a negatively charged monolayer of the H-EPSNa membrane, designed to be highly ultrasound resistant and emit a strong ultrasound signal through contraction and expansion. [6–8]

**Fig. 2:**
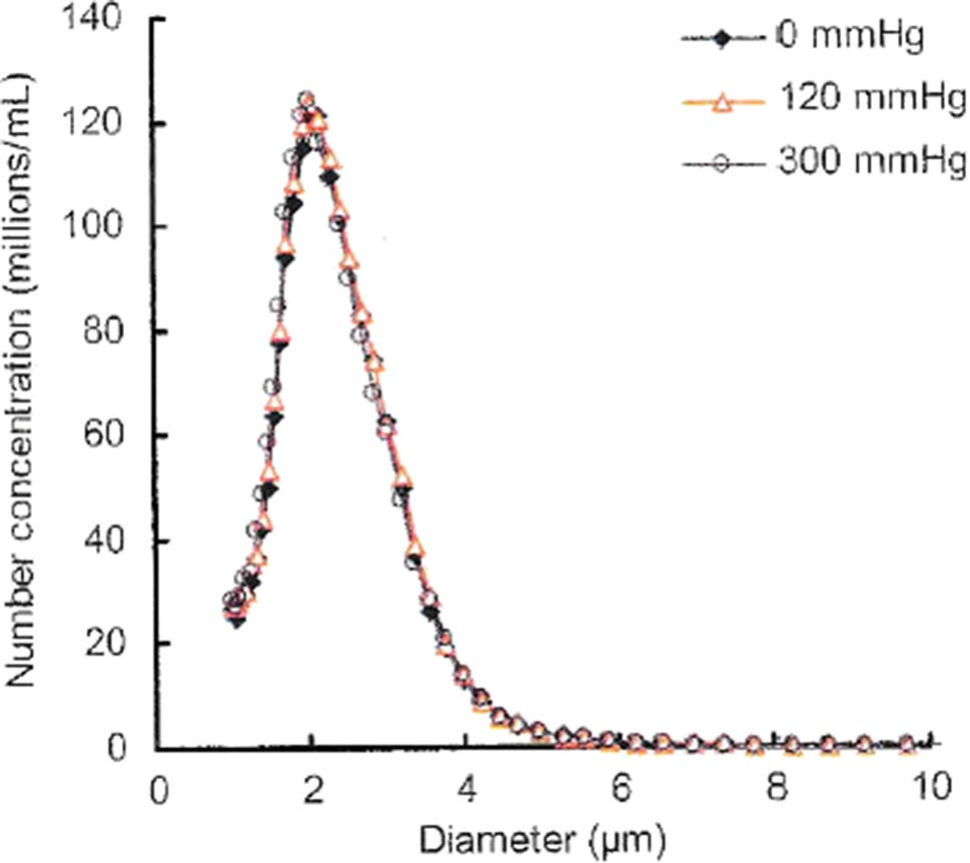
Effect of increased hydrostatic pressure on the microsphere content and size distribution of Sonazoid reconstituted product. Samples have been pressurized for 60 s at stated pressure before release and re-analysis. Adapted from Ref. [8]

The (commercial) formulation is provided as a lyophilized injection. The PFB microbubble is embedded in amorphous purified white sugar, which is dissolved when 2 mL of the supplied water for injection is added, yielding the PFB microbubble suspension. The PFB microbubble has a particle size of 2.3–2.9 µm (mean particle size), which is smaller than that of erythrocytes. It thus can easily pass through pulmonary capillaries and circulate throughout the body after intravenous administration. Transmission electron microscopy revealed a shell thickness of 2–3 nm. The particle size distribution of the drug is shown in Fig. 2 and is controlled so that fractions larger than 7 µm are kept as low as possible [8].

## Acoustic properties of Sonazoid

One of the fundamental acoustic properties of the PFB microbubble is backscatter. The backscatter signal is the echo back to the probe and is used to obtain images during clinical use. Although backscatter is a more direct parameter in assessing efficacy, its relationship to the size of the PFB microbubble is difficult to measure, so it is calculated based on theoretical considerations.

**Fig. 3:**
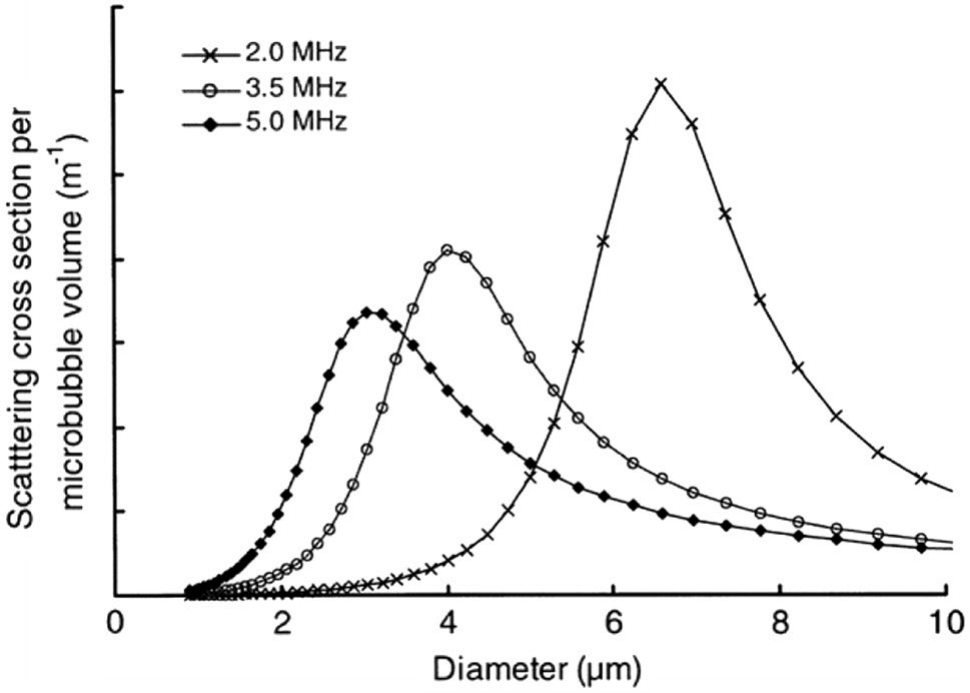
The theoretical scattering efficacy (scattering cross-section) per microbubble volume of NC100100 versus microbubble diameter at various frequencies. Adapted from Ref. [9]

The relationship between backscattering intensity and particle size is calculated from the Kelvin–Voigt theoretical model, and the particle size at which the backscattering intensity is optimal increases with decreasing transmitted frequency. The optimum particle size of the PFB microbubble is 3–7 µm (Fig. 3) [9] since the transmitted frequencies of 2–5 MHz used in the study are typically the same frequency range as in clinical practice. Although probes with higher frequencies are used in the mammary region, the average particle size of Sonazoid is approximately 2–3 µm, which should not be a problem for use.

## Administration and imaging methods for Sonazoid

Three different doses of Sonazoid were compared in phase II clinical trials. The middle dose of 0.015 mL/kg (body weight) was found to have the best contrast enhancement effect, and this dose became the standard volume [3–5]. Although Sonazoid is usually administered through a peripheral blood vessel, the elbow vein is chosen to increase the peak concentration in the blood after intravenous infusion. Since the volume of Sonazoid solution administered IV is small (less than 1 mL), the peak blood concentration can be increased by flushing with 10–20 mL of saline solution. Usually, an extension tube is used for intravenous bolus infusion, filled with Sonazoid solution and flushed with 10–20 mL of saline solution. Strong pressure should not be applied to the Sonazoid solution in the extension tube. If pressure is applied above a certain level, the air bubbles in the tube will disappear instantly.

The probe should be a linear probe for the body surface that supports contrast-enhanced ultrasound. The choice of sound pressure and imaging method is important. The optimum sound pressure depends on the contrast agent. For Sonazoid, an mechanical index (MI) value of around 0.2 is recommended as an index of sound pressure. The filter, pulse inversion, and amplitude modulation methods are known as contrast mode imaging methods, with the amplitude modulation method mainly being used for Sonazoid mammograms. The optimal frequency depends on the imaging method, but frequencies around 5–8 MHz are used. The focus should be set deeper than the center of the lesion to obtain a contrast effect over a wider area. Sound pressure, imaging methods, and contrast effects are explained in the next section.

## Harmonic signals generated by microbubbles

**Fig. 4:**
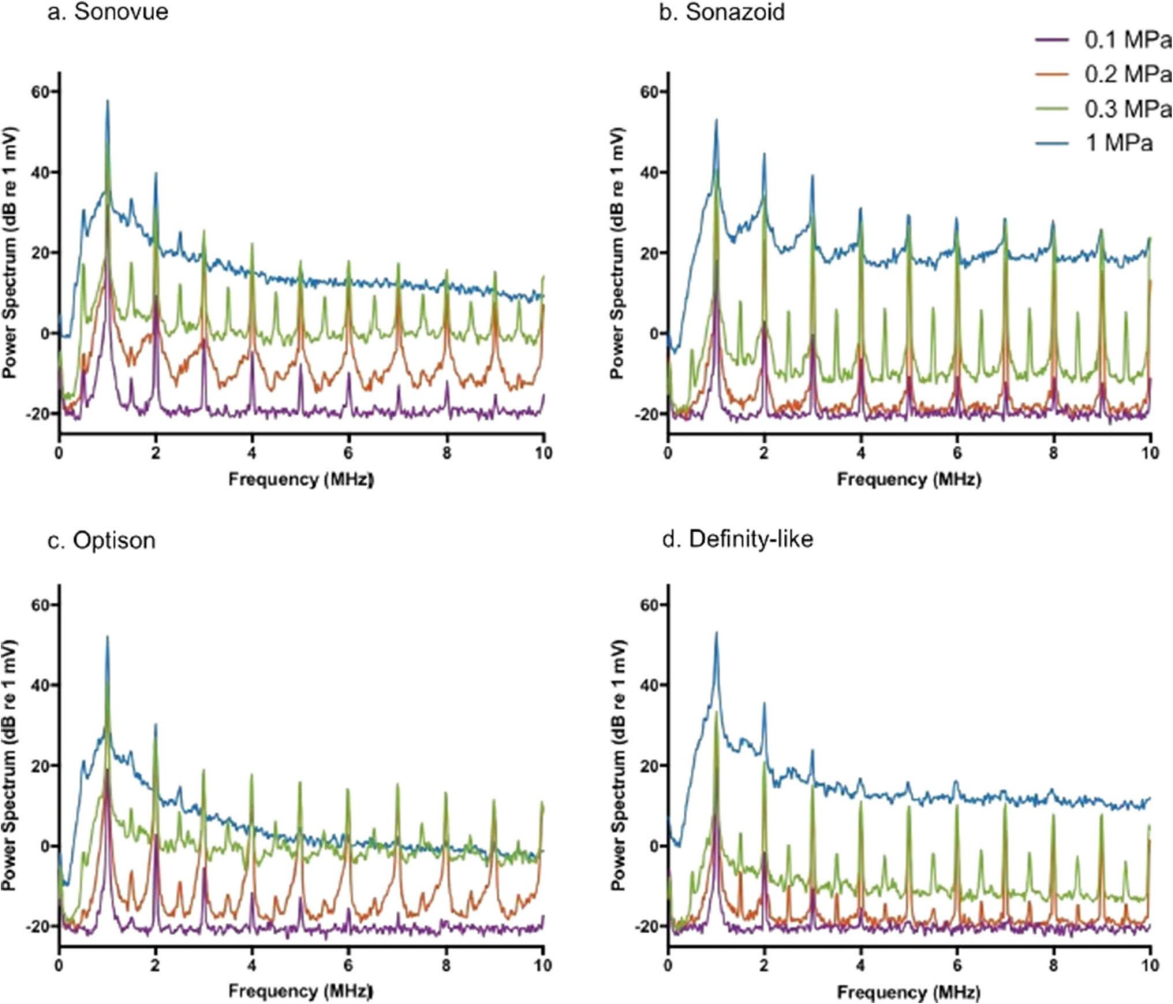
Average frequency spectra of 50 microbubble echoes at different excitation pressures received using the C308 single element transducer from the four agents used: **a** Sonovue, **b** Sonazoid, **c** Optison, and **d** “Definity-like”. Adapted from Ref. [11]

It is known that microbubbles, the main component of ultrasound contrast media, resonate upon ultrasound irradiation and have nonlinear harmonic components. Kono et al. reported that the characteristics of the microbubble depend on the composition of the microbubble of the ultrasound contrast agent [10]. Keller et al. measured the frequency characteristics of Sonovue, Sonazoid, Optison, and a custom “Definity-like” agent [11]. Figure 4 shows the frequency response of the echo signal from each microbubble when the sound pressure increases from 0.1 to 0.2 MPa, 0.3 MPa, and 1 MPa for 1-MHz ultrasound transmitted with a 40-cycle pulse length. It can be observed that as the sound pressure increases, harmonics (2 MHz, 3 MHz, 4 MHz, etc.) are generated and increase for the fundamental wave (1 MHz). Sonazoid maintains a strong harmonic signal at high sound pressure.

Low MI is usually selected for continuous real-time imaging to minimize microbubble disruption. In the guidelines for the liver [12], the manufacturer’s recommendation of 0.06 is selected as the optimal MI for Sonovue when using the iU22 ultrasound system (Philips), and 0.2 is recommended for Sonazoid when using the Logiq 7 device (GE). Sonazoid has a stiffer shell and requires a higher acoustic amplitude than Sonovue to generate nonlinear vibrations.

Ultrasound contrast imaging is called contrast harmonic imaging (CHI), where nonlinear harmonic components from microbubbles are detected and imaged in order to eliminate the linear echo components from the surrounding tissue in the fundamental band.

## Harmonic signals generated by tissue

Ultrasound contrast imaging is performed by separating the echo signal produced by microbubbles from the echo signal of the surrounding tissue. To achieve this, it is first necessary to know the characteristics of the echo signal of the tissue.

**Fig. 5:**
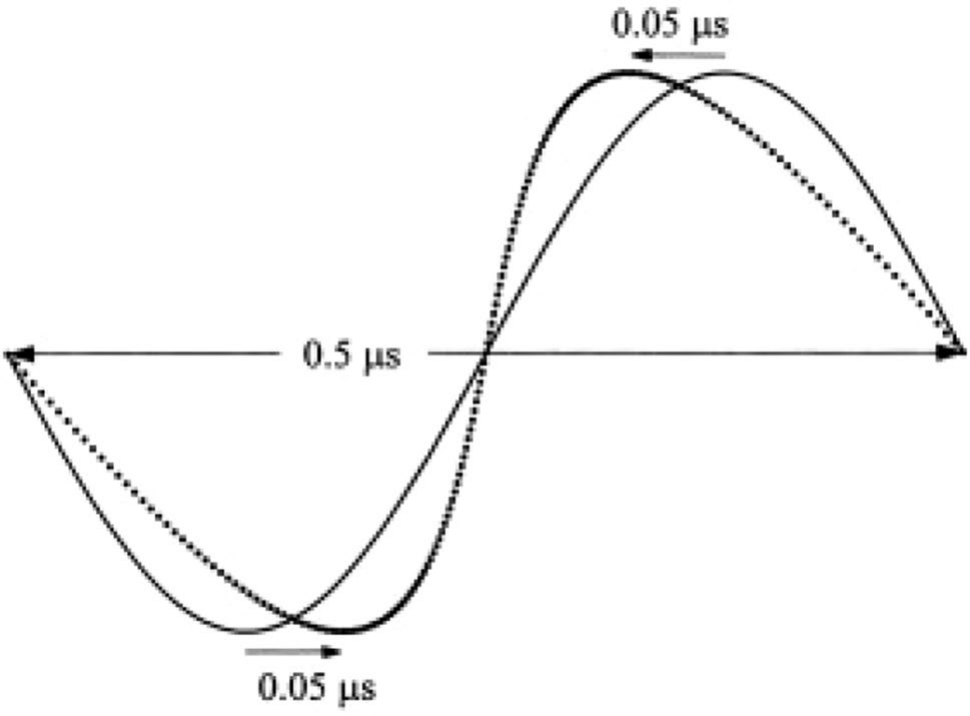
Waveform distortion of a 2-MHz plane wave with an amplitude of 10^5^ Pa at a distance of 0.5 m. Adapted from Ref. [14]

**Fig. 6:**
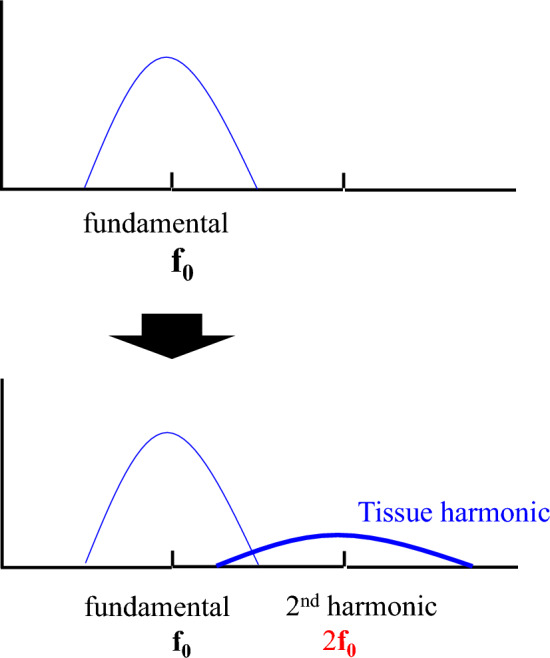
Spectra of waveform distortion

The propagation velocity of ultrasonic waves increases depending upon the pressure applied to the propagating medium. The speed of sound in water is said to be about 1500 m/s. However, in the ocean, for example, the speed of sound is quite different between deep water (where high hydrostatic pressure is applied) and water near the sea surface (where the pressure is almost atmospheric). It is known that the speed of sound increases by 0.16 m/s with an increase in water depth of 10 m (an increase in water pressure of 1 atm = 10^5^ Pa). This relationship between water pressure and sound speed also applies to the relationship between sound pressure and sound speed. Figure 5 shows an example of nonlinear waveform distortion during ultrasonic propagation caused by the sound pressure dependence of sound velocity. The figure shows the waveform distortion (dashed line) for an ultrasonic wave with an amplitude of 10^5^ Pa at 2 MHz when propagating 50 cm. The speed of sound at 10^5^ Pa, the positive maximum value, is 1500.233 m/s, which is faster by only 0.233 m/s. At this time, the waveform is distorted by 0.05 μs, as shown in Fig. 5 [13]. This corresponds to a large waveform change at one-tenth of a single cycle at 2 MHz, and as shown in the schematic diagram in Fig. 6, a second-harmonic component is generated. Such a phenomenon is nonlinear propagation of ultrasound, and the harmonic component is called tissue harmonic [13–19]. Therefore, even if the waveform of the sound source is sinusoidal, the sound pressure waveform after propagation changes, as shown in Fig. 5. The waveform change is cumulative with ultrasonic wave propagation, and its degree depends on the product of ultrasonic pressure and propagation distance. Tissue harmonic imaging (THI), which visualizes tissue using the nonlinear component of this propagation, is used in diagnostic ultrasound systems. THI has a narrow main beam and lower side lobes, leading to improvement in spatial and contrast resolution, respectively. In CHI, however, the tissue harmonic components lower the detection sensitivity of harmonic components derived from contrast agents.

## Signal processing to separate microbubble and tissue signals

When imaging the microvasculature of tissue using the contrast echo method, the echo signals from the surrounding tissue around the capillaries are strong, so the conventional B-mode method that uses the fundamental frequency component does not provide sufficient contrast. The main component of ultrasound contrast agents is microbubbles, and by focusing on the nonlinear characteristics of bubbles, ultrasound diagnostic systems have adopted methods to remove echo signals from the surrounding tissue and visualize only nonlinear signal components from the contrast agents.

**Fig. 7:**
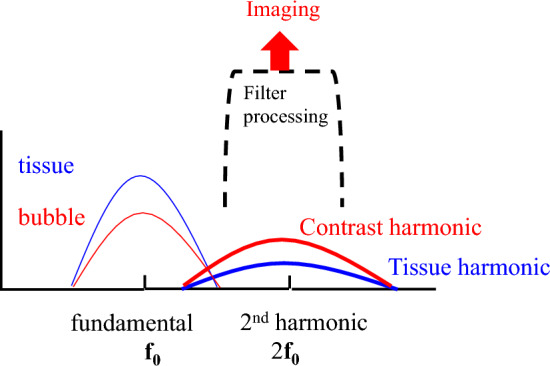
Spectra of waveform distortion

Figure 7 shows a schematic diagram of the frequency spectrum of the received signal from the tissue and the received signal from the contrast agent. At the fundamental frequency (*f*_0_), the echo component from the tissue exceeds that from the contrast agent. The B-mode of this fundamental component cannot visualize the contrast agent. However, in the second-harmonic component (2*f*_0_), which is twice the fundamental frequency (*f*_0_), the harmonic component of the contrast agent is larger than the tissue harmonic component due to tissue propagation. Therefore, the second-harmonic component (2*f*_0_) can visualize the contrast agent [20–22].

The filter and pulse inversion methods are described as methods to remove echo components from the surrounding tissue in the fundamental band and to visualize nonlinear signal components from the contrast agent in the second-harmonic band. We then describe the amplitude modulation and amplitude-modulated pulse inversion methods, which significantly remove the tissue component in the fundamental band and detect and visualize only the nonlinear signal component from the contrast agent [23, 24]. The amplitude modulation method plays an essential role in Sonazoid contrast in the mammary gland, and we will review technical papers on the mechanism of this method and discuss it in detail.

## Filter method

The filter method was the first CHI technique to visualize nonlinear signal components from the contrast agent. As shown in Fig. 7, the filter method removes the echo signal from the surrounding tissue that dominates the fundamental frequency band and detects and visualizes the nonlinear signal component from the contrast agent in the second-harmonic band.

Fundamental frequency components dominate echo signals produced by tissues, and the harmonic components are small. The harmonic components generated by microbubbles, which vibrate well even at low sound pressures, are far larger than those of tissue. As shown in Fig. 7, the filter method can detect and visualize the echo signals of microbubbles. Miyatake et al. showed that microvascular flow in dog myocardium can be visualized on real-time second-harmonic grayscale images using the filter method with Levovist, whose gas component is air, under low sound pressure conditions [21]. Moriyasu et al. showed that both Levovist and Optison, which uses carbon fluoride as a gas component, can visualize the dynamic enhancement of dog liver from the hepatic artery to the portal vein and liver parenchyma on real-time second-harmonic grayscale images under low sound pressure conditions [22]. Furthermore, Kono et al. reported enhancement patterns in localized lesions in the liver [25]. Burns et al. applied second-harmonic detection to power Doppler to visualize blood flow through microvessels in the kidney [26]. Figure 7 shows an overlap between the bandwidth of the fundamental component (*f*_0_) and the nonlinear second-harmonic component (2*f*_0_). The frequency bandwidth of the ultrasonic pulse should be narrowed to eliminate the overlap. It means increasing the length of the ultrasound pulse. The disadvantage of the filter method is decreased spatial resolution. On the other hand, the filter processing is performed within a single ultrasound transmission/reception. The advantage is to provide high temporal resolution. The filter method is used in the CHI mode in the cardiovascular field, where real-time performance is essential [21].

## Pulse inversion method (PI)

**Fig. 8:**
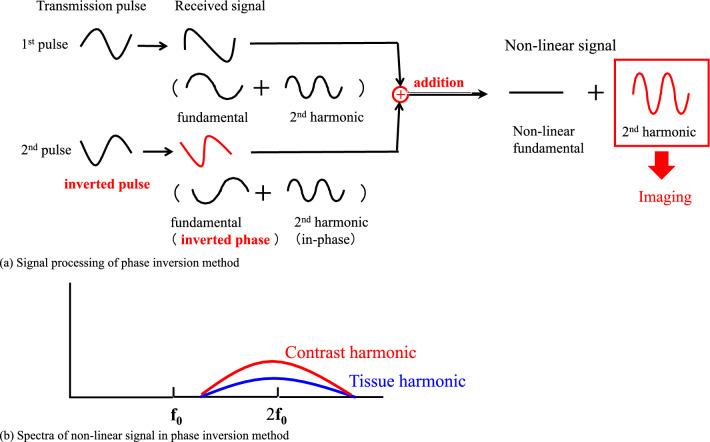
Pulse inversion method

The pulse inversion method has been developed to visualize second-harmonic components in a wide bandwidth. Figure 8 shows a conceptual diagram of the signal processing of the pulse inversion method. Figure 8a shows bubbles are irradiated with phase-inverted ultrasonic waves once each, and the received signals are added and processed. Since the fundamental frequency component in the received signal is similar to the transmitted waveform, its phase is also inverted according to the transmitted waveform. However, the nonlinear second-harmonic components are in phase. Only the nonlinear second-harmonic components remain when the two received signals are added.

Figure 8b shows the spectrum of the received signal after the addition process. The two transmitted pulses have the same amplitude and opposite phase components in the fundamental band. In the received signal, both the tissue and the microbubble signal have the same amplitude and opposite phase components in the fundamental band. Therefore, when the two signals are added, the signals cancel in the fundamental band, as shown in Fig. 8b. On the other hand, in the second-harmonic band, both the harmonic signals from the tissue and the microbubble are added in-phase and extracted. In the pulse inversion method, these second-harmonic components are used to generate the contrast image.

Since the fundamental component can be removed with signal processing, nonlinear signal components in the second-harmonic band can be extracted even if the fundamental and second-harmonic signals overlap. It allows us to obtain an image with good spatial resolution using the broadband second-harmonic components.

In this technique, the THI component of the tissue is not removed but detected. However, in the contrast-enhanced mode, the sound pressure is low enough to avoid the destruction of the microbubbles, and the THI component is somewhat suppressed. As shown in the figure, since the nonlinearity is more significant for bubbles than tissue, the microbubble signal exceeds the tissue signal in the second-harmonic band, allowing the microbubble distribution to be imaged [27, 28].

Up to this point, the tissue harmonic component due to tissue propagation has been reduced but not eliminated. Tissue harmonic images are depicted in the image, and weak enhanced areas may not be visible. The amplitude modulation and amplitude-modulated pulse inversion methods are described as methods to detect and visualize only the nonlinear signal components derived from the contrast agent by removing the tissue component.

## Amplitude modulation method (AM)

**Fig. 9:**
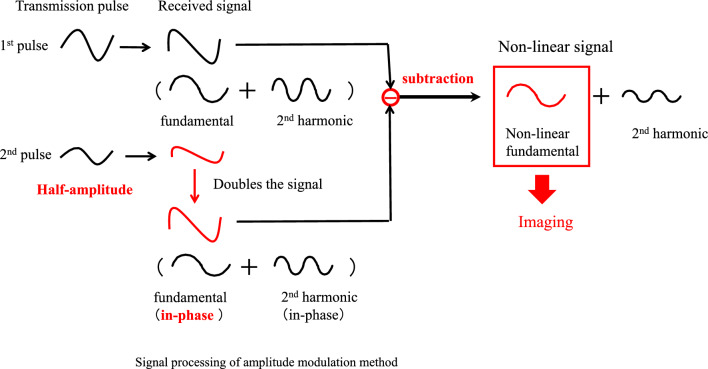
Amplitude modulation method

Since the nonlinear propagation of tissue and expansion/contraction of microbubbles generate harmonic components, there is a limit to separating these phenomena by simple filtering or pulse inversion methods that focus on the difference in frequency bands. Figure 9 shows a conceptual diagram of signal processing for the amplitude modulation method. The microbubble is irradiated with ultrasound waves of different amplitudes once each. The fundamental frequency components in the echo signal are similar to the transmitted waveform, so the received signal is proportional to the transmitted amplitude. The lower panel of Fig. 9 shows the second transmission and reception waveforms. Since the transmitted amplitude is multiplied by 1/2, the received signal is also multiplied by 1/2. The amplitude of the second received signal is multiplied by 2. The amplitudes are aligned and then subtracted from the first received signal. The linear fundamental components are then canceled out. However, the amplitude of the nonlinear component is not proportional to the transmitted amplitude due to its sound pressure dependence. The nonlinear components do not cancel out and remain in the difference. The amplitude modulation method can extract the nonlinear components in the fundamental and second-harmonic bands from the received signal. The nonlinear components in the second-harmonic band are the harmonic signals from the tissue and microbubble, but the difference processing reduces the signal. The signal in the fundamental band is the nonlinear component from the microbubble. The mechanism of generation of this component is described below. As with the pulse inversion method, only the filter can extract the nonlinear signal in the second-harmonic band. However, as with the pulse inversion method, the problem of the included THI component arises. The amplitude modulation method focuses on the nonlinear component of the contrast agent in the fundamental band. The nonlinear tissue component in the fundamental band is considered to be small. The simulation results are discussed below. As shown in Fig. 9, by extracting the signal in the fundamental band with a filter, only the signal from the contrast agent is extracted, and a contrast image is generated [23, 29–35].

## Amplitude-modulated pulse inversion method (AMPI)

**Fig. 10:**
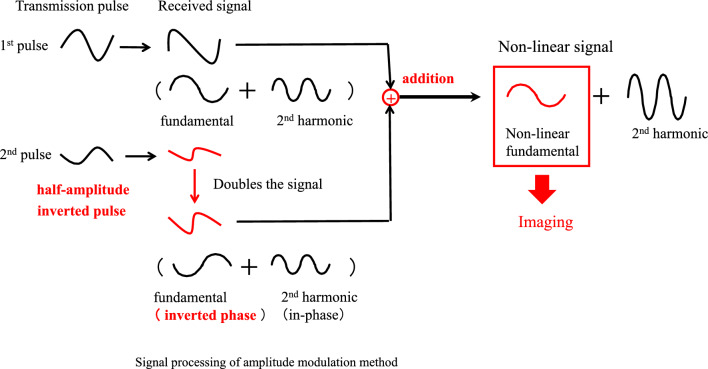
Amplitude-modulated pulse inversion method

The amplitude-modulated pulse inversion method has also been proposed, combining the pulse inversion and amplitude modulation methods described above. Figure 10 shows a conceptual diagram of the signal processing of the amplitude-modulated pulse inversion method. In the second transmission, the phase is inverted, and the amplitude is changed. At the reception time, nonlinear signals can be extracted by adding these two echoes after correcting their amplitudes.

The upper part of Fig. 10 shows the first transmission and reception waveforms. The lower part of Fig. 10 shows the second transmission and reception waveforms. Since the phase is inverted and the transmitted amplitude is multiplied by 1/2, the received signal is also inverted in phase, and its amplitude is multiplied by 1/2. The second received signal is multiplied by 2, and its amplitude is aligned and then added to the first received signal. The linear fundamental component is canceled. However, the amplitude of the nonlinear component is not proportional to the transmitted amplitude due to its sound pressure dependence. The nonlinear components are not canceled out, and a difference remains. The amplitude-modulated pulse inversion method can extract the nonlinear components in the fundamental and second-harmonic bands from the received signal. The signals in the fundamental band are nonlinear components from microbubbles. As with the amplitude modulation method, we focus on the nonlinear contrast agent component in the fundamental band. As shown in Fig. 10, only the signal from the contrast agent is extracted, and a contrast image is generated by utilizing the fundamental band signal with a filter [23, 29, 30, 36–38].

## Simulation-based study of signal processing (PI, AM, AMPI)

The effects of phase inversion, amplitude modulation, and amplitude-modulated pulse inversion methods are studied by simulating the scattered echoes of microbubbles. Models are used to formulate microbubble behavior [39–47]. When the pressure around the microbubble changes, the bubble repeatedly contracts and expands in the radial direction, forming a spring-mass system that oscillates with nonlinearity.

Nonlinear oscillations of shell-less bubbles are modeled by the Rayleigh–Plesset equation [44].

Second-generation microbubble contrast agents such as Sonazoid are microbubbles with a shell of protein or lipid around the bubble and filled with insoluble gas. Formulations of the scattered echoes of shelled microbubbles have also been studied [46–50].

Below is a model by Morgan et al. [51, 52] in which the effects of the shell elastic term and viscosity of lipid shell bubbles are considered:

1 $$\rho R\ddot{R}+\frac{3}{2}\rho {\dot{R}}^{2}=\left({P}_{0}+\frac{2\sigma }{{R}_{0}}+\frac{2\chi }{{R}_{0}}\right){\left(\frac{{R}_{0}}{R}\right)}^{3\gamma }\left(1-\frac{3\gamma }{c}\dot{R}\right)-\frac{4\mu \dot{R}}{R}-\frac{2\sigma }{R}\left(1-\frac{1}{c}\dot{R}\right)-\frac{2\chi }{R}{\left(\frac{{R}_{0}}{R}\right)}^{2}\left(1-\frac{3}{c}\dot{R}\right)-12{\mu }_{sh}\epsilon \frac{\dot{R}}{R\left(R-\epsilon \right)}-\left({P}_{0}+{P}_{{\text{driv}}}\left(t\right)\right),$$

2 $${P}_{{\text{driv}}}\left(t\right)={P}_{a}{\text{exp}}\left\{-\pi {\left(\frac{t-{t}_{0}}{{T}_{g}}\right)}^{2}\right\}{\text{sin}}\left\{2\pi {f}_{0}\left(t-{t}_{0}\right)\right\}.$$

This includes the contributions of the shell elasticity, χ; shell viscosity, µ_sh_; liquid viscosity, µ; and the interfacial tension, *σ*.

The experiments presented in this paper were performed using the contrast agent Definity, whose bubble shell is a lipid shell, and Morgan et al. obtained the elastic and viscous terms of the lipid-shelled bubble shell by fitting the simulation results to the experimental data. The parameters used in the simulations are the values used by Morgan et al. [51, 52].

Shell parameters used in the final fits were *χ* = 0 N/m and *µ*_sh_ = 0.6 nm Pa s.

The shell thickness was set to *ϵ* = 1 nm; the Sonazoid shell is a monolayer of sodium hydrogenated egg yolk phosphatidylserine (H-EPSNa) with a shell thickness of 2–3 nm. The results of this simulation are likely to be in error with respect to the behavior of Sonazoid. On the other hand, in clinical use, both Definity and Sonazoid show similar characteristics for various contrast methods. We think that the simulation and experimental results of the lipid shell bubbles are useful for understanding the characteristics of Sonazoid.

The predicted received echoes are formulated as follows:

3 $$P\left(R, t, r\right)=\frac{\rho }{r}\left({R}^{2}\ddot{R}+2R({\dot{R})}^{2}\right).$$

Sato et al. studied the extraction of nonlinear components of microbubbles [29–31] in each imaging method by simulating the pulse inversion, amplitude modulation, and amplitude-modulated pulse inversion methods using the model of Morgan et al. [51]. The values for the model parameters are given in Table 1. The transmitted pulses used were of six different types, including three with different peak sound pressures (100 kPa, 200 kPa, and 400 kPa) and two with initial phases of 0° and 180°:

**Table 1:** Parameters used in evaluation of model

*c*	Speed of sound in liquid	1540	m/s
*f*_0_	Frequency of incident acoustic field	2.5	MHz
*P*_0_	Hydrostatic pressure	101	kPa
*ϵ*	Thickness of lipid shell	1	nm
*γ*	Polytropic gas exponent	1.07	
*μ*	Viscosity	0.001	Pa・s
*ρ*	Liquid density	998	kg/m^3^
*σ*	Interfacial tension coefficient	0.051	N/m
*χ*	Elasticity modulus of lipid shell	0	N/m
*μ*_sh_	Viscosity of lipid shell	0.6	nm・Pa・s
Pa		360	kPa
Tg		2/f_0_	

P_1_: Sound pressure 100 kPa, initial phase 0°, P_2_: Sound pressure 100 kPa, initial phase 180°.

P_3_: Sound pressure 200 kPa, initial phase 0°, P_4_: Sound pressure 200 kPa, initial phase 180°.

P_5_: Sound pressure 400 kPa, initial phase 0°, P_6_: Sound pressure 400 kPa, initial phase 180°.

**Fig. 11:**
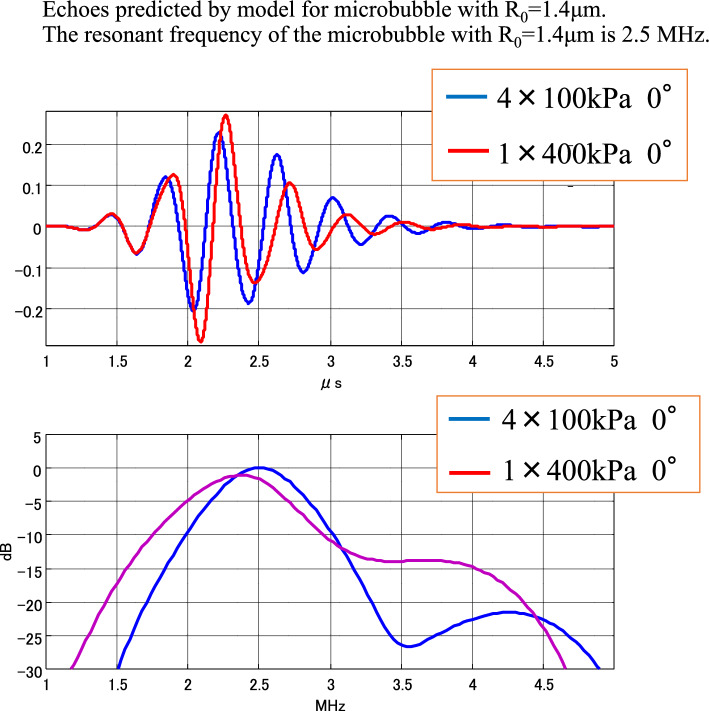
Waveform and spectra of predicted echoes

Figure 11 shows the predicted echo results for different sound pressures. In the case shown as an example, the transmitted frequency and the resonant frequency of the bubble are almost equal. Predicted echoes at a peak sound pressure of 100 kPa are shown by blue lines, and those at a peak sound pressure of 400 kPa by red lines. Only the amplitudes of the sound pressure waveforms are corrected based on the applied sound pressures to be aligned.

Increasing the sound pressure not only causes nonlinearity in the amplitude but also changes the phase and causes a delay. In the spectrum, the peak frequency decreases as the sound pressure increases. It can be seen that a change in ultrasonic transmitted pressure changes not only the amplitude of the predicted echoes but also their frequency and phase.

The predicted received signal described above can be used to simulate the phase modulation (PI), amplitude modulation (AM), and phase amplitude modulation (AMPI) methods. Using Eqs. (4) to (6), the received echoes from *P*_1_ to *P*_4_ can be combined to generate the received signal for each imaging method as follows:

4 $${\text{PI}} = P_{{3}} + \, P_{{4}} ,$$

5 $${\text{AM }} = \, P_{{3}} - { 2}P_{{1}} ,$$

6 $${\text{AMPI }} = P_{{3}} + { 2}P_{{2}} .$$

**Fig. 12:**
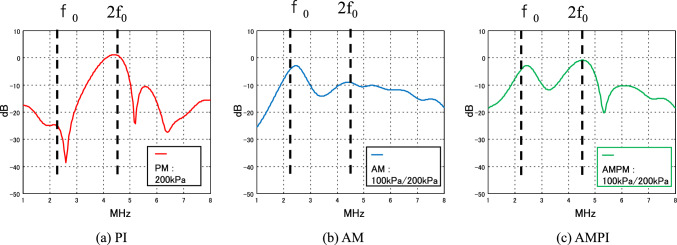
Simulation of spectra of predicted echoes with three pulse sequences (PI, AM, AMPI)

Figure 12 shows the simulation results when the transmitted frequency and the resonant frequency of the bubble are almost equal.

Figure 12a shows the frequency response of the PI method. A nonlinear component in the second-harmonic band is detected. Figure 12b shows the frequency response of the AM method. The detection of nonlinear components in the second-harmonic band (2*f*_0_) is smaller than that of the PI method. However, the fundamental band has a signal component (*f*_0_). It is not a linear component of the fundamental band but a nonlinear component from the microbubble. This signal is called nonlinear fundamental. It is detected by the change in frequency and phase with the change in sound pressure shown in Fig. 11. It is a nonlinear component that cannot be detected with the PI method, which does not change the sound pressure of the transmitted ultrasonic wave.

Figure 12c shows the frequency response of the AMPI method, which has the characteristics of both the PI and AM methods. It detects nonlinear components in the second-harmonic band (2*f*_0_) and large nonlinear components in the fundamental band (*f*_0_). Nonlinear signals in the fundamental band in AM and AMPI have been reported by Averkiou et al. [23] in a simulation using a simple bubble dynamics model (the Gilmore equation) [44]. Keller et al. [53] have shown that microbubble echoes exhibit amplitude-dependent nonlinearity and an amplitude-dependent difference in phase in the AM method. Tremblay-Darveau et al. [54] posited that an amplitude-dependent difference in phase results from the buckling dynamics of shelled microbubbles, which was demonstrated with the Marmottant model [50].

## Experimental study of signal processing (PI, AM, AMPI)

**Fig. 13:**
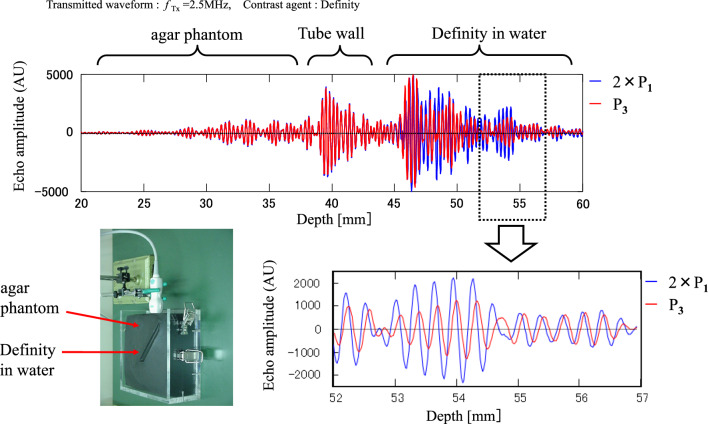
Experimental echoes with three pulse sequences (PI, AM, AMPI)

As with the simulation, four types of ultrasound waves with different amplitudes and phases were transmitted to an agar phantom in Fig. 13. The echoes in water with the contrast agent Definity were analyzed. The transmitted frequency is 2.5 MHz, MI = 0.1.

*P*_1_: MI = 0.05, initial phase 0°,

*P*_2_: MI = 0.05, initial phase 180°.

*P*_3_: MI = 0.1, initial phase 0,

*P*_4_: MI = 0.1, initial phase 180°.

As with the simulations, the received echoes from *P*_1_ to *P*_4_ were combined using Eqs. (4) to (6) to generate the received signal for each imaging method.

As shown in Fig. 13, the waveforms from the agar phantom and tube wall do not show phase and amplitude changes with sound pressure. However, the waveforms from Definity in water show that the phase and amplitude change with sound pressure.

*P*_1_ is shown in blue, and *P*_3_ in red. The blue color (*P*_1_) represents a condition where the sound pressure is half that of the red (*P*_3_). The amplitude of *P*_1_ is doubled, and the magnitudes of *P*_1_ and *P*_3_ are aligned. It is observed that the phase changes as the sound pressure changes. Similar results to the simulation have been obtained experimentally. The phase change of the reflected signal of the contrast agent is considered to be the mechanism of nonlinear signal generation in the fundamental band.

**Fig. 14:**
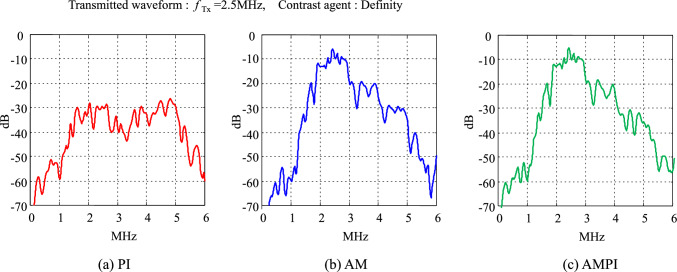
Spectra of experimental echoes with two pulse sequences (PI, AM)

Figure 14 compares the characteristics after processing the PI method, the AM method, and the AMPI method. Similar to the simulation results, the red plot in Fig. 14a shows the frequency response of the PI method, which detects nonlinear components in the second-harmonic band (2f_0_). The blue plot in Fig. 14b is the frequency response of the AM method, which detects strong nonlinear components in the fundamental band (*f*_0_). The green plot in Fig. 14c is the frequency response of the AMPI method, which also detects strong nonlinear components in the fundamental band (*f*_0_). A nonlinear component called “nonlinear fundamental” was also observed in the experiment. It is caused by changes in frequency and phase when the sound pressure is changed, as shown in Fig. 13. Eckersley et al. have reported similar findings using Definity [38]. In this experiment, the second-harmonic component from the contrast agent was smaller than the level expected in the simulation.

When choosing one of the above imaging methods for ultrasound contrast in the mammary gland, it is essential to consider the specific characteristics of Sonazoid. The optimum transmitted conditions for Sonazoid are high around MI 0.2, which tends to generate THI components. Sato et al. confirmed in experiments using Definity that the nonlinear component of tissue propagation in the fundamental band is smaller than the THI component in the second-harmonic band [30]. We can expect better enhancement of the Sonazoid echo signal in the fundamental band than that in the second-harmonic band around MI 0.2.

Keller et al. have studied in detail that microbubble echoes exhibit amplitude-dependent nonlinearity, and amplitude-dependent phase differences occur in the AM method. Using the Rayleigh–Plesset equation [44] and the Marmottant equation [50], simulations of non-shelled microbubbles and shelled microbubbles are used to evaluate amplitude-dependent phase differences (⊿φ_AM_). For tissue-derived nonlinear signals, nonlinear propagation in tissue is simulated using the Khokhlov–Zabolotskaya–Kuznetsov (KZK) equation. It has been shown that the magnitude of⊿φ_AM_ is much smaller for nonlinear propagation than for microbubbles.

**Fig. 15:**
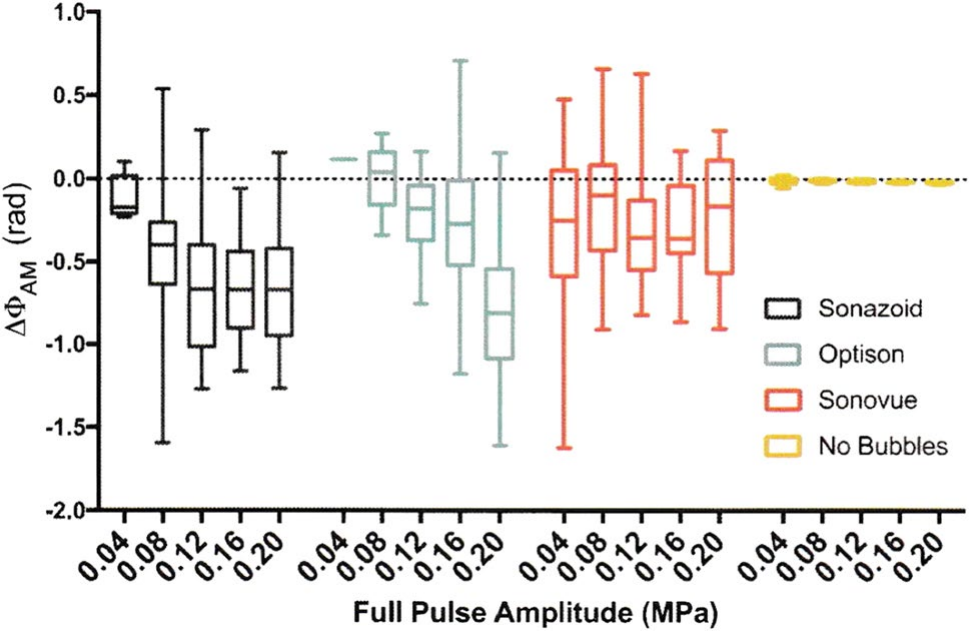
⊿φ_AM_ measurements from scattering experiments. All microbubble types produced a negative ⊿φ_AM_, while linear echoes from a metal tube did not produce obvious ⊿φ_AM_, although a very slight decline registered at the highest pressures. Adapted from Ref. [53]

Three commercially available ultrasound contrast agents, Optison, Sonazoid, and Sonovue, were used to measure ⊿φ_AM_ in experiments. The results are compared to a linear reflector without microbubbles [53] and are shown in Fig. 15. ⊿φ_AM_ was observed for all microbubbles, consistent with theoretical results. There was no apparent ⊿φ_AM_ due to scattering from the linear reflector.

Thus, it is suggested that the advantage of the AM and AMPI methods is the ability to eliminate THI components in the second-harmonic band and the infrequent occurrence of amplitude-dependent phase differences due to tissue origin in the fundamental band. In order to observe enhancement with Sonazoid in the breast with high sensitivity, the AM and AMPI methods, which can eliminate tissue-derived nonlinear components, are considered beneficial.

## Conclusion

We explained the principles of the filter, pulse inversion, amplitude modulation, and amplitude-modulated pulse inversion methods as imaging methods used in contrast-enhanced ultrasonography. The pulse inversion method, which visualizes the second-harmonic component using the nonlinear scattering characteristics of the contrast agent, is widely used regardless of the contrast agent or target organ. Sonazoid, which is used as a contrast agent for the mammary gland in Japan, has a recommended transmission condition of around MI 0.2, and its higher transmitted sound pressure than other contrast agents, such as Sonovue, generates a significant THI component. The pulse inversion method also detects the THI component, which reduces contrast sensitivity. The AM and AMPI methods detect nonlinear signals from the contrast agent in the fundamental band. The mechanism is thought to be a phase change of the contrast agent echo signal depending on the sound pressure. Simulations and experiments suggest that nonlinear signals from tissue in the fundamental band are small. The AM and AMPI methods are considered suitable for contrast-enhanced ultrasonography of the breast with Sonazoid.
