# Correction: Principle of contrast-enhanced ultrasonography

**DOI:** 10.1007/s10396-024-01500-5

**Published:** 2024-09-21

**Authors:** Yoshitaka Mine, Etsuo Takada, Katsutoshi Sugimoto, Fuminori Moriyasu

**Affiliations:** 1 Department of Radiological Sciences, International University of Health and Welfare, 2600-1 Kitakanemaru, Otawara, Tochigi 324-8501 Japan; 2 Center of Medical Ultrasonics, Dokkyo Medical University, Mibu, Tochigi Japan; 3 Department of Gastroenterology and Hepatology, Tokyo Medical University, Tokyo, Japan; 4 Center for Cancer Ablation Therapy, Sanno Hospital, International University of Health and Welfare, Tokyo, Japan

**Correction: Journal of Medical Ultrasonics** 10.1007/s10396-024-01443-x

The article Principle of contrast‑enhanced ultrasonography, written by Yoshitaka Mine, Etsuo Takada, Katsutoshi Sugimoto and Fuminori Moriyasu, was originally published electronically on the publisher’s internet portal on 23 May 2024 without open access. With the society’s decision to opt for Open Choice the copyright of the article changed on 29 August 2024 to © The Author(s) 2024 and the article is forthwith distributed under a Creative Commons Attribution 4.0 International License, which permits use, sharing, adaptation, distribution and reproduction in any medium or format, as long as you give appropriate credit to the original author(s) and the source, provide a link to the Creative Commons licence, and indicate if changes were made. The images or other third party material in this article are included in the article’s Creative Commons licence, unless indicated otherwise in a credit line to the material. If material is not included in the article’s Creative Commons licence and your intended use is not permitted by statutory regulation or exceeds the permitted use, you will need to obtain permission directly from the copyright holder. To view a copy of this licence, visit http://creativecommons.org/licenses/by/4.0.

The original article has been corrected.

